# Robotics in Lower-Limb Rehabilitation after Stroke

**DOI:** 10.1155/2017/3731802

**Published:** 2017-06-08

**Authors:** Xue Zhang, Zan Yue, Jing Wang

**Affiliations:** School of Mechanical Engineering, Xi'an Jiaotong University, Xi'an 710049, China

## Abstract

With the increase in the elderly, stroke has become a common disease, often leading to motor dysfunction and even permanent disability. Lower-limb rehabilitation robots can help patients to carry out reasonable and effective training to improve the motor function of paralyzed extremity. In this paper, the developments of lower-limb rehabilitation robots in the past decades are reviewed. Specifically, we provide a classification, a comparison, and a design overview of the driving modes, training paradigm, and control strategy of the lower-limb rehabilitation robots in the reviewed literature. A brief review on the gait detection technology of lower-limb rehabilitation robots is also presented. Finally, we discuss the future directions of the lower-limb rehabilitation robots.

## 1. Introduction

Stroke is an illness that has a high potential of causing disability in the aged [1]. With the increase in the elderly, stroke has become a common disease, which often leads to motor dysfunction or even permanent disability [2]. There are about 795,000 people in the United States each year, and about 191,000 people in Japan who have had a new stroke or recurrent stroke [3]. The number of new stroke patients in China is about 200 million each year [4]. According to the national stroke statistics, stroke morbidity, mortality, and recurrence rate increase with age [5]. At the same time, stroke incidence showed a younger trend in recent years. As a result, the rehabilitation training of stroke survivors has become a major social problem urgently. However, traditional manual therapies such as physical therapy (PT) and occupation therapy (OT) mainly depend on the experience of the therapist, and it is difficult to meet the requirements of high-intensity and repetitive training [6]. Due to the serious shortage of physiotherapists, the treatment cannot be guaranteed [7]. As a result, the demand for advanced rehabilitation equipment is significantly increasing, which will help patients to perform accurate, quantitative, and effective training [8]. Rehabilitation robotics is an emerging field expected to be a solution for automated training. Over the past decade, rehabilitation robots received increasing attention from researchers as well as rehabilitation physicians. The application of rehabilitation robot can release the doctors from heavy training tasks, analyze the data of the robot during the training process, and evaluate the patient's rehabilitation status. Due to the advantages of their accuracy and reliability, rehabilitation robots can provide an effective way to improve the outcome of stroke or postsurgical rehabilitation.

Nowadays, there have been several published review papers on lower-limb rehabilitation robot. However, very few details of control strategies, driving modes, training modes, and gait perception were given to the lower-limb rehabilitation robot.

In this paper, we systematically reviewed the current development of lower-limb rehabilitation robot, providing a classification, a comparison and a design overview of the driving modes, training paradigm, control strategy, and gait perception. The rest of the paper is organized as follows.  Section 2 described the development of robots.  Section 3 introduced the driving modes of the lower-limb rehabilitation robot.  Section 4 presented control strategies, including position control, force signal control, and biological medical signal control. In Section 5, the training pattern of the robot was recommended. In Section 6, different techniques of the gait perception were analyzed. In Section 7, limitations of the study and future direction of development were discussed and summarized.

## 2. Development of Lower-Limb Rehabilitation Robots

In recent years, various types of lower-limb rehabilitation robots have been developed to enhance the motor function of paralyzed limbs in stroke patients. In general, lower-limb rehabilitation robots can be divided into two categories, that is, exoskeleton robots and end-effector robots [9]. For example, Lokomat [10], BLEEX [11], and LOPES [12, 13] are typical exoskeleton robots, while Rutgers Ankle [14] and Haptic Walker [15] are end-effector robots. According to their rehabilitation principles, exoskeleton robots can be divided into treadmill-based and leg orthoses, while the end-effector robots have footplate-based and platform-based types. An overview of recent representative robots and their characteristics are demonstrated in Table 1.

### 2.1. Treadmill-Based Exoskeleton Robots

The Lokomat, LokoHelp, Lopes, and Active Leg Exoskeleton (ALEX) belong to the typical treadmill-based exoskeleton robots. Treadmill-based exoskeleton robots are usually composed of a weight support system and runs on a treadmill through the lower-limb exoskeleton frame.

In 2001, the Swiss Federal Institute of technology in Zurich [33] developed the four freedom exoskeleton type gait rehabilitation robot Lokomat, with the use of treadmills. The exoskeleton can drive the leg of the patient to realize the gait motion in the sagittal plane, and the four rotary joints are driven by four DC motors to drive the precision ball screw transmission.

LokoHelp is a gait-training robot, which was developed and produced by a German company, consisting of three parts, a leg brace device, treadmill system, and suspension weight system. It can achieve the basic gait rehabilitation training and help patients complete the downhill exercise. In addition, the equipment adopts a modular design method, which is easy to assemble, disassemble, and adjust, in order to realize the training of different slope. Clinical experimental studies on LokoHelp have proved that [34, 35] the rehabilitation effect of the robot system is almost the same as that of the traditional gait training method, but it significantly reduces the required human resources and the physical exertion of the participants.

The Biomedical Engineering Laboratory at the University of Twente [36], Holland, has developed a lower extremity-powered exoskeleton gait rehabilitation robot (LOPES) [37, 38]. A LOPES single leg has 2 degrees of freedom in the hip joint and 1 degree of freedom in the knee joint. LOPES divided the patient's recovery into two stages: patient dominant and robot driven, and different control algorithms are used to make the walking training of the patients closer to the actual situation.

The School of Mechanical Engineering, Delaware University, has developed an active walking training robot called ALEX. It consists of a moving bracket, lower extremity exoskeleton orthosis, and a control system. Each leg has four degrees of freedom, two degrees of freedom of the hip joint, and one degree of freedom of knee and ankle joints. The back of ALEX, using mechanical mechanisms to balance the gravity of the human body, can help patients achieve gravity balance and altitude adjustment [39, 40].

### 2.2. Leg Orthoses and Exoskeletons

The Active Ankle-Foot Orthosis (AAFO) [41], Knee-Ankle-Foot Orthosis (KAFO), Berkley Lower Extremity Exoskeleton (BLEEX), and Hybrid Assistive Limb (HAL) belong to the leg orthoses and exoskeletons.

Yonsei University, Seoul, Korea, developed a single degree of freedom hinge ankle-foot orthoses AAFO. The orthosis uses a polypropylene material, which is lightweight and has a certain degree of flexibility. Moreover, the joint uses a hinge structure; the driving part adopts the series elastic actuator. The contact between the foot and the ground is determined by installing a contact switch on the foot [42] and using the plantar state machine on the ankle foot orthosis control. The gait is divided into 6 phases to prevent foot drop in foot slap orthosis and toe drag stage [43].

In 2004, Dr. H. Kazerooni of the University of California-Berkeley [44] designed the lower-limb exoskeleton robot BLEEX (Berkeley Lower Extremity Exoskeleton), and designers called it “weight-bearing and energy independent exoskeleton.” According to the force of the exoskeleton, the inverse dynamic model of the exoskeleton is used as the feedforward controller and the joint angle sensor is used to judge the movement period of each leg and control the coordinated movement of the exoskeleton. Through the experimental study of four patients with paraplegia, the exoskeleton robot can help patients achieve natural walking [45].

In 2005, the Department of Mechanical Engineering of the Ottawa University [46] in Canada developed the Knee-Ankle-Foot Orthosis (KAFOs), to help users of weak extensor improve the gait. This orthosis does not use drive and provides the power with the ingenious mechanical structure and the position of the spring, and it controls the flexion and extension of the knee joint through opening and shutting off the solenoid. The robot control system is simple, and it mainly uses the plantar force to control on-off solenoid and complete assist standing control.

Hybrid Assistive Limb (HAL) is a wearable lower-limb rehabilitation robot developed by the University of Tsukuba, Japan. The original purpose of the device was to assist patients with lower-limb motor dysfunction to complete the routine activities such as walking, standing, sitting, and going up- and downstairs [47]. At present, a fifth generation of the products has been developed, a whole body wearable robot, which can assist the upper and lower limb movement [48]. Notably, some clinical and experimental studies showed that HAL can provide weight support for the subjects and can help them complete their daily walking activities.

### 2.3. Foot Plate-Based End-Effector Devices

The foot plate-based end-effector devices [49] consist of the Gait Trainer GTI, Haptic Walker, and the G-EO Systems.

Gait Trainer (GTI) is a suspension weight loss gait rehabilitation robot, developed by the Free University Berlin, Germany. It was based on the movement of the lower limb to stimulate the muscles of the lower limb orderly and assist the patient to complete gait training. However, because of the interaction between the foot pedal and the patient's foot, the force feedback of the lower limbs was weak, and the feeling of walking was larger than that of natural walking. In addition, the robot's gait training strategy emphasized repetitive passive motion, while ignoring the importance of active participation. GTI was an early device for lower-limb rehabilitation, and there were many clinical trials in the world [50–54]; the system reduces the physical strength consumption significantly and also saved the medical resources for rehabilitation.

In 2003, Hesse et al. proposed the concept of Haptic Walker based on virtual reality technology. They developed as a foot motion simulator 6 degrees of freedom, with the use of hanging weight loss to realize the arbitrary trajectory and the attitude motion in the sagittal plane, such as walking on the rough surface or the lawn, tripping, and so forth. In the virtual reality control mode, the patient wore a helmet display and a six-degree-of-freedom force sensor was installed on the foot pedal; the patient felt the virtual reality scene and interacted with the virtual scene. The virtual scene and music can also improve the monotonous training atmosphere and enhance the training interest of patients, to achieve the purpose of psychotherapy. The virtual walking rehabilitation training robot was the first device to realize the foot walking along the programmable free trajectory, and redundant hardware and software emergency stop circuits were set up on security as measures.

Compared to other sports platform, a robot actuated by foot in Italy is driven by the pedal with lower-limb movement. The robot added a new way of walking, such as obstacle, step, and slope road. The training rich mode and the active and passive control mode can be a more effective targeted training [55]. The computer comes with a huge data integration system, which can monitor the patient's rehabilitation index in real time. This robot uses pedal structure, which is very comfortable and is easy to use for the patient. However, due to the lack of auxiliary devices in the legs, the patient's muscle strength is too strong or too weak to get the appropriate adjustment, so a doctor is also needed from the side to help [56].

### 2.4. Platform-Based End-Effector Robots

The Ruegst ankle, ARBOT, and parallel ankle robots belong to the platform based on end-effector robots.

The first truly fully used for ankle rehabilitation robot system was the “Rutgers ankle” proposed by Girone et al. of Rutgers University [57]. It was a robot system based on the Stewart platform [58] with virtual reality, force feedback, and remote control [59]. The mechanism was composed of a fixed platform, a movable platform, and six telescopic branched chains that were connected with the movable platform. It could carry out six independent movements with 6 degrees of freedom. The Stewart platform used six double acting cylinders to drive six degrees of freedom motion, and the virtual reality based human-computer interactive game provided by the host makes the training process no longer boring. Through the received data, doctors could understand the movement of the ankle joint, and then use the network to control, evaluate, and guide the patients to carry out the appropriate rehabilitation training.

In comparison, exoskeleton robots are usually fixed in various parts of the human limb, while producing different forces/torques. However, for different patients, these exoskeleton robots may not be able to restore the patient's limb function due to its disadvantages and poor adaptability. The end-effector robot is usually at a certain point in contact with the patient's body. Because there is no restriction on the movement of human, the end effector is easier to adapt to different patients [60].

## 3. Driving Mode of Lower-Limb Rehabilitation Robots

The choice of driving mode directly effects the system scheme of the exoskeleton robot, such as structure design and control system, and it is the basis of exoskeleton robot design. At the moment, the common drive modes of an exoskeleton robot are hydraulic drive, motor drive, pneumatic drive, and SEA (series elastic actuator) [61, 62]. There are other drive modes, such as pneumatic muscle and electronic rod. We summarize different driving modes in Table 2.

Nowadays, the rehabilitation exoskeleton robot mostly used motor drive mode; the robot just needs to bear the body weight and assist hemiplegic patients in common activities, such as walking and going up and down the stairs. Compared with other drive modes, motor drive mode has many advantages, like easy control, no pollution, low noise, and so forth. Hydraulic drive mode is much simpler, smaller, and lighter than other modes. Under the same load, the hydraulic drive is much better than the other driving methods.

In summary, the drivers, such as hydraulic, motor, pneumatic, and SEA (series elastic actuator) are limited by the power, mass, and volume, and the consequence of noise on people in the work is serious. Although the development of artificial muscles plays an important role in the problems, there are some technical challenges to overcome. Another important aspect is the drivers' energy problem. The usable energy, such as nonrechargeable battery, rechargeable battery, and small internal combustion engine, has both merits and limitations, so the potential and perpetual method to solve these problems is to develop new technologies, like electrochemical fuel cell and wireless energy transmission.

## 4. Control Strategies for Lower-Limb Rehabilitation Robots

According to the different signals that are obtained from the initiative intention, the control strategy between robot and patients is divided into three parts:

1. Position control
2. Force signal control
3. Biological medical signal control.

### 4.1. Position Control

The position control method is trajectory-tracking control, which is to drive the lower limbs to walk on the fixed mode. The gait is formed by a proportional position feedback controller and joint angles and suitable for lower limb muscle strength. Hornby confirmed the efficacy of trajectory tracking control, which can increase the speed and durability of patients with incomplete spinal cord injury. Zhang et al. established the trajectory tracking control of the 5 connection model, which can enhance the participation of the patients and make the training more personalized [73].

### 4.2. Force Signal Control

In this control strategy, force signal is produced by limb contraction and interactions with mechanical structure. The interaction force can be directly measured by force and moment sensor in the elegant mechanical structure design, which can be evaluated by the kinetics models of the human-computer interactive system. Compared with biological medical signal, force signal has a better determinacy, which can better reflect the motion intention of the patient, so the control based on force signal is feasible and relatively steady. However, the acquisition of interaction force usually requires mechanical structure, which is less available than biological medical signal detection, so the applicable range of interactive controlling are limited. In the interaction control strategy, between rehabilitation robot and patient, there are two most widely used methods: hybrid force/position control and impedance control [74].

#### 4.2.1. Force/Position Hybrid Control

To resolve the control problems of robot in a constrained environment [75], Raibert proposed the force/position hybrid control strategy. Sometimes, we should control the position of the robot on some specific directions, but on the other directions, we should control the interaction force between the mechanical structure and the outside world. Therefore, when the robot contacts the outside world, the task space of robot would be split into two subspaces in the force/position hybrid control strategy. The subspaces are position subspace and force subspace, and it will complete the tracking control over position and force in the corresponding subspace [76]. The interaction control of lower-limb rehabilitative robot is aiming to provide a safe, comfortable, and flexible place for treatment and healing, and it does not need accurate force trace control, so force/position hybrid control strategy is uncommonly used in interactive controls.

Lokomat achieved a new cooperative gait training strategy by using force/position hybrid control method [77]. The control of lower limb gait orthosis is a two-stage process. In the step stage, according to the dynamic model to control the power of the orthosis to provide reasonable support for patients, it is difficult to accurately assess the relevant kinetics model. Therefore, we just control the position of orthosis in the standing stage. Besides that, gait stages of the limbs are monitored in real time, as the converting signals of hybrid control in the two stages. This strategy can help patients to walk freely, and it requires active and full engagement of the limbs of patients. Therefore, it is an active rehabilitation training, which is highly intention-oriented and stimulates the patients to participate positively and with initiative in the rehabilitative training; it will accelerate the recovery process.

#### 4.2.2. Impedance Control

Impedance control is different from force/position hybrid control. It focuses on realizing the flexibility of the rehabilitation robot, which avoids excessive force between the mechanical structure and limbs. This method could provide a natural, comfortable, and safe touch interface and avoid secondary damage effectively. An additional advantage of impedance control is that the achievement of impedance control is independent of the prior knowledge [78]. In the control of interaction force between robot and patients, impedance control has a more extensive application.

In the robot control field, the theory of impedance control was first proposed by Hogan [79], and it was the spread of damping control and rigidity control. Seen from the approach of realization, impedance control can be divided into two categories: one is based on torque and the other is based on position. The first one is based on forward-facing impedance equations, but the explicit expressions of impedance equations do not exist in the control structures generally. The second one is based on reverse impedance equations, which is also called admittance control. It usually adopts a typical double closed-loop control structure; the outside loop controls the force and the inner loop controls the position. The impedance control based on position is easier to realize [80, 81] position servo control, more mature, and stable. Aiming at Gait Trainer (GTI) of lower-limb rehabilitative robot, Hussein proposed an adaptive impedance control algorithm for gait training [82].

### 4.3. Biological Medical Signal Control

Surface electromyogram (sEMG) and electroencephalogram (EEG) are mostly used in interactive controlling of lower-limb rehabilitative robot. Since these signals are both using nonintrusive ways to get, the ways of obtaining the sEMG and EEG are operable and do not need a medical expert and its performance can get guarantees.

#### 4.3.1. The Control Based on sEMG

EMG signal is the electrical activity produced by the skeletal muscle [83, 84]. According to different measurement methods, it is mainly composed of sEMG and iEMG (intramuscular EMG). sEMG is a signal obtained by attaching electrodes to the surface of the skin, while iEMG is a signal obtained by inserting a needle electrode into the muscle tissue beneath the skin. Compared with the active signal, sEMG has the following advantages:

1. The acquisition of sEMG is simple and does not require a complex mechanical structure design.
2. The force signal is just the embodiment of all muscle groups, and sEMG can reflect the degree of activity of specific muscle groups, which can be more detailed monitoring and control of the movement of the limbs.
3. The interactive control based on sEMG has more flexibility, which can realize the control of the healthy limb to the diseased limb according to the coordination of the body movement.
4. sEMG has higher sensitivity and resolution than the active force signal, and it is more suitable to use sEMG to detect active motion intention for the patients with lower limb autonomy.

The challenges of interactive control method based on sEMG are as follows. First, through the human skin, to collect sEMG signal has great randomness, and in order to obtain the signals, which have high signal-to-noise ratio and can truly reflect the muscle activity, we need to find an effective way to filter out the interference of sEMG. Secondly, the single channel sEMG only reflects the activity of specific muscles, in order to obtain the active motion intention of the patients, which is usually necessary to combine multiple muscle activities. In contrast, the response of the force signal to the active intention is more direct.

The interactive control strategy based on sEMG can be divided into two categories:

1. Using the remaining EMG of diseased limbs. This method can not only stimulate the patients' awareness of active participation but also encourage patients to control the contraction of limb muscles during exercise. But for severely paralyzed patients, their diseased limbs have almost completely lost their motor function and cannot complete muscle contraction independently; the sEMG signal is so weak that it is difficult to be detected. The first scheme is not applicable in this case.
2. Using the motion coordination of the left and right limbs or upper and lower limbs and EMG signals of the healthy limbs control the movement of the paralyzed limb. This method in active participation of patients is less than the first strategy, but it provides an active training program for severely paralyzed patients.

#### 4.3.2. The Control Based on EEG

The EEG signal is the electrical activity of the brain [85], which is collected by electrodes attached to the scalp, and it represents the voltage fluctuations caused by the flow of ions between the neurons in the brain.

The most important advantage of interactive control based on EEG is that it is limited to the extent of physical disability; even if the patient has completely lost the motor function of the lower limb, as long as the brain can produce motion control signals, the method is equally applicable. This method is particularly suitable for patients with complete spinal cord injury, and their brain function is normal, but the control signal transduction pathway is cut off, so the muscles of the limbs completely lost control. The interactive control based on EEG is equivalent to the reconstruction of the brain control signal transmission path outside the body, and the motor and functional electrical stimulation device are used as the actuator to regain the control of the limb motor function.

This method is limited to the paralyzed patients whose brain motor control function is normal, but it is not suitable for patients with brain damage caused by stroke and other reasons, because the brain motor function area of the patients has been damaged and it has not been able to produce the EEG signal of normal limb movement control. Secondly, compared with the sEMG signal, the resolution of EEG on limb movement intention is low and the EEG signal has a greater randomness, in which changes in expression, mood, and attention will easily effect the EEG signal generated by the brain.

At present, the research in this area is mainly focused on offline classification knowledge and regression analysis; the knowledge pointed out the potential of the interactive control of lower-limb rehabilitation robot based on the EEG, but the actual application and the experimental results are almost none. Compared with offline research, real-time interactive control is facing more challenges. First, the real-time acquisition of the EEG signal is not possible to have the integrity of the data used in offline research; the accuracy of the identification may be affected. Secondly, it is necessary to ensure the real-time performance of interactive control, which requires the use of EEG signal for motion recognition, more importantly, to predict. Finally, in real-time interactive control, the patient will not be able to complete the actual physical movement independently, the acquisition of the EEG signal corresponds to the movement of the brain, and this has not been considered in the study of the existing lower-limb rehabilitation robot.

## 5. Training Modes of Lower-Limb Rehabilitation Robot

**Figure 1:**
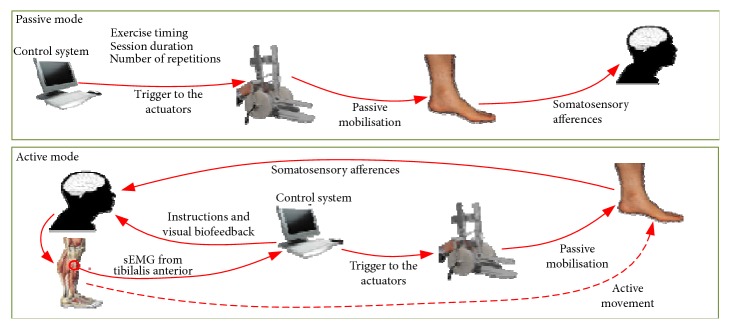
Passive and active control modes [88].

The effectiveness of lower-limb rehabilitation robots and treatment depends largely on its training mode [86], which will assist the patient in different patterns of movement according to the patient's recovery [87].  Figure 1 shows two typical control modes for rehabilitation robots: passive mode and active mode [89]. Recently, more subdivided training modes for lower-limb rehabilitation have been proposed. An overview of modes for rehabilitation robot is illustrated in Table 3.

The rehabilitation-training mode is divided into four kinds, which includes the passive mode, the active assist mode, active mode, and active resist mode.

In the passive mode, the patient lost muscle strength and could not complete the active movement. We can only rely on the help of external forces to achieve the patient's passive training. The robot's legs drive people's legs for rehabilitation training, and the lower-limb rehabilitation robot should provide sufficient strength for passive training. The advantage of this model is through the repeated exercise to promote the recovery of limb motor function and reduce muscle atrophy, but the patient lacks motivation.

In the active mode, the muscles of the patient have certain strength and the active motion of the smaller torque can be performed on the rehabilitation equipment. When the patient wants to move his joint or limb, the robot device will use an external assist force as needed. It requires the robot to perceive the state of the patient and the force/torque when following the patient's movement. This model can be modified according to the patient's intention, thereby greatly enhancing the initiative of patients.

In the active assist model, the muscles have certain strength, but without the help of the robot legs, patients cannot be fully trained. This allows the patient to move without the help of a robot, which can improve the patient's ability to exercise independently.

In the active resistance model, the mechanical leg provides a certain force, which is opposite to the direction of the leg to achieve the purpose of strengthening muscle training. This model is suitable for patients with high recovery, and resistance makes the movement more challenging and can enhance muscle strength in patients.

At present, there are a number of other training methods, such as mirror motion and isotonic and isokinetic exercise patterns. Although these new training patterns are similar from the therapist's view, they are also trying to provide assistance or resistance to the patient in the course of robotic therapy.

## 6. Gait Detection Technology

Accurate signal is the foundation of control; the quantitative feedback information is helpful for developing reasonable rehabilitation strategy according to the state of patients. Therefore, the choosing of sensor, which can detect the information of human computer interaction, is crucial. Gait detection technology consists of three primary parts: plantar sensing technology, limb sensing technology, and mixed sensing technology.

Plantar sensing technology: it can judge the different gaits by detecting the man-machine forces or the ground reaction forces of foot using sensor.

Limbs sensing technology: it uses sensors to detect motion intention of the lower limbs or the torso:

1. The sensing technology based on angle sensor
2. The sensing technology based on EMG [96] sensor
3. The sensing technology based on BCI [97].

Mixed sensing technology can be applied to identify and judge the human gaits using two or more sensors together.

Combining of the detection information of all kinds of sensors, the control system can obtain accurate movement information to make sure that the exoskeleton robot will work effectively and reliably.

At present, there are two main ways for detecting motion intention, as shown in Table 4. One is human robot interaction based on physical models (pHRI) [100]. It is mainly used for detecting interaction information between patient and exoskeleton, such as position information, force information, and so forth. Although there are some kinds of lag in time, and the sensors' installations effect the comfort ability, this method is of high reliability. The other is human robot interaction based on cognition (cHRI) [100]. Using this method, motion intention of patients, as input signals for controller, is gained through the identification of EMG [96] signals. Patching the sensors on the skin directly is very comfortable, but the sweat on the skin can seriously effect measurement precision, and it also cannot ensure the one-to-one mapping relationship between the EMG signals and the joint torque. At the same time, the misjudgments of the controller can cause secondary damage. Obviously, we can accurately judge for motion intention by fusing the two kinds of signals. The detection method of human robot interaction information is presented in Table 4.

The exoskeleton rehabilitation robot uses many kinds of sensors to detect gait, but the detection methods still have many problems, such as vulnerability to interference, inaccurate judgment, and poor adaptability. Therefore, the development of BCI technology and sensor technology are crucial to solve the current problems.

## 7. Discussion

In this paper, the development of lower-limb rehabilitation robot, training mode, driving mode, control strategy, and gait detection technology are reviewed. The lower-limb rehabilitation robot has many advantages, and it has shown encouraging clinical outcomes and rehabilitation efficiency. Although most of the lower-limb rehabilitation robots can provide systematic and long-term treatment, there are still some disadvantages and deficiencies summarized as follows:

1. The mechanical structure and control system of rehabilitative robot need to be improved. During rehabilitation training, it lacks exact control in real time for patients' joints angles, torque, speed, etc.
2. The recently developed robots in domestic and abroad are mainly on motor rigid drive. The system is lacking in flexibility, and the actuator structure of rehabilitation robot is overly complex and large with low portability. At the same time, the security and comfort also need further improvement.
3. The feedback mechanism of rehabilitative effect should be consummated. It could not give an accurate feedback to the limbs' position and force during rehabilitation training, which causes low training efficiency and directly effects the evaluation of rehabilitation training.
4. For a flexible robot, we need to develop a more advanced high polymer as flexible material. Moreover, the driving force still needs to be improved.
5. Patients' motivation involved in the training plays a very important role in stroke rehabilitation. However, most training paradigms are rigid and boring. Task-oriented training paradigm with interesting games such as whack-a-mole can make the training more enjoyable.
6. The lower-limb rehabilitation robot still faces numerous technological challenges, including the biomechanics, neurophysiology, human-computer interaction (HCI), and ergonomics.

The current lower-limb rehabilitation robots, to some extent, can provide a simple training program for patients and has a certain effect on rehabilitation. In our opinion, future researches on lower-limb rehabilitation robot should focus on the following aspects:

1. System design of lower-limb rehabilitation robot: the mechanical structural design is the foundation of robotic system, which needs to achieve some major objectives, such as compact, multi-DOF, great flexibility, various kinds of training methods and motions, better comfort, and high matching between human and computer.
2. The control strategies and motion pattern design of lower-limb rehabilitation robot: due to the individual difference of the patients, the robot should perceive state information of patient's force and position, to adopt corresponding training mode and control strategy. Future researches, such as adaptability and stability of control system, the applications of sensor technique, and the design of control algorithm, are required. Therefore, the robot should not only meet the demand of low weight, fast response, and large output torque but also have some characteristics similar to animal skeleton muscles, such as pliability and reliability. Therefore, it is important to research the optimizing design method for energy saving based on active and passive mode, the energy technology of high energy density, and wireless transmission technology.
3. The design of gait detection system: the lower-limb rehabilitation robot should be able to detect and perceive the information of interaction forces and motion position between the patient and rehabilitation robot. On the one hand, the robot should provide appropriate assistance, when the patient could not complete motion by himself. On the other hand, the robot should decrease the assist force or increase the resistance properly, when the motor ability of paralyzed lower extremity improves remarkably.
4. Security protection mechanism: the robot must be designed to meet the safety requirements of clinical rehabilitation training, while preventing damage. In order to ensure security of rehabilitation training, two important issues should be considered when designing the lower-limb rehabilitation robots: mechanism design (hardware) and control system (software).
5. Rehabilitation effect assessment system: by combining the detection of EMG signals and EEG signals. We should explore the inherent relationship between the rehabilitation effectiveness and the train parameters and develop new assessment strategies to verify the effectiveness of the lower-limb rehabilitation robot.
6. The VR technology has been proved to be an effective tool in neurorehabilitation. On the one hand, the interesting and varied virtual scene in VR improves more motivation of patients comparing with the training course in traditional training. On the other hand, the immersive VR environment can effectively stimulate human brain mirror neurons in the motor cortex and promote the recovery of the nerve. However, VR cannot provide physical feedback to the paralyzed limb; the robot can compensate for this defect. Therefore, the combination of rehabilitation robot and VR technology is the future development direction. However, before the application, the following core issues must be addressed:
  - The exact factors in the design of VR, which stimulate patients' motor cortex mirror neurons, should be explored in the future.
  - The vertigo problem of VR, which limits the application of VR system, must be solved.

## Figures and Tables

**Table 1:** Overview of recent lower-limb rehabilitation robots.

Groups	Devices	Researchers	Actuated DoF	Driving modes	Control strategies	Training modes
	Lokomat [101]	Zurich Switzerland	Two-leg DoFs	Motor drive	Position control Patient-cooperative strategy Posture control	Passive mode Active assist mode
	LokoHelp [16]	Woodway & LokoHelp Group	Two-leg DoFs	Treadmill drive, standalone driving device not required	Trajectory tracking control	Passive mode Active assist mode
Treadmill-based exoskeleton robots	ALEX [17]	Banala and Aqrawal et al. from University of Delaware, US	Seven DoFs for translations and rotation of a leg	Motor drive	Assist-as-needed control	Active mode
	Lopes [18, 19]	Reneman et al. From university of Delaware, US	Three rotational DoFs in each leg	SEA (series elastic actuator) drive	Impedance control	Active mode Active assist mode
	AAFO [20]	Seoul and Korea from Yonsei University	Two motion DoFs for ankle joint	SEA (series elastic actuator) drive	Force/impedance control	Active mode
	KAFO [21]	The Department of Mechanical Engineering of the Ottawa University	Free motion DoFs in sagittal plane for ankle and knee	No driver, using the location of mechanical structure and spring to provide tailwind	Force control	Active assist mode

Leg orthoses exoskeleton robots	HAL [22]	University of Tsukuba, Japan	Full-body exoskeleton for arms, legs	Motor drive	Autonomous control Automatic mixture control	Active assist mode
	BLEEX [23, 24]	Kazeroom et al. from University of California, US	Seven DoFs for each leg in hip, knee, and ankle joints	Hydraulic drive	EMG signal control Force control	Passive mode
	Rutgers ankle [25]	Girone et al. of Rutgers University	Six DoFs ankle and foot based on a Stewart platform	Pneumatic drive	Impedance control Force control	Active mode Passive mode Active resist mode

Platform-based end-effector robots	ARBOT [26, 27]	Saglia et al. from Istituto Italiano di Tecnologia, Italy	Two ankle DoFs in plantar/dorsiflexion, inversion/eversion	Motor drive	Position control	Passive mode Active assist mode Active resist mode
	Parallel ankle robots [28, 29]	Xie et al. from the University of Auckland New Zealand	Three ankle DoFs provided by 4-axis parallel robot	Motor drive	EMG-based evaluation and adaptive control	Active mode Passive mode
	Gait Trainer GTI [30]	The Free University Berlin, Germany	Two footplates for foot/leg movement	Motor drive	Trajectory tracking control	Passive mode Active mode

Footplate-based end-effector robots	Haptic Walker [31]	Hesse et al. from Charite University Hospital, Germany	Arbitrary movement DoFs for two feet	Motor drive	Trajectory tracking control	Passive mode Active mode
	G-EO Systems [32]	Reha Technology AG, Switzerland	Two footplates for walking and climbing DoFs	Motor drive	Position control Trajectory tracking control	Active assist mode

**Table 2:** Overview of driving modes for rehabilitation robot.

Drive types	Definition	Advantages	Disadvantages	Representative works
Hydraulic drive [63–65]	Taking the liquid as the actuating medium for energy transmission and control	(1) High reliability (2) Simple structure (3) Working stability (4) Low inertia (5) The overload protection is easily realized (6) It can realize stepless speed regulation.	(1) It is sensitive to oil temperature and loading change (2) The hydraulic oil can be compressed (3) The working fluid is easy to leak; high noise; low energy efficiency; low drive speed.	BLEEX series, University of California Berkeley, US

Motor drive [66–68]	Using electric equipments and adjusting the circuit parameters for power transmission and control	(1) The cable for connection has advantages of energy transfer convenient, signal transform quickly (2) High level standard (3) Easily to achieve automatic control (4) Simple structure (5) Nonpolluting.	(1) It has poor balance of movement (2) It is easily influenced by external load (3) Large inertia (4) Slow change (5) Large volume (6) Heavy.	HAL series, Tsukuba University of Cyberdyne, Japan

Pneumatic drive [69–71]	Taking the compressed air as the actuating medium for energy transmission and control	(1) Simple structure (2) Low cost (3) Small gas viscosity (4) It can realize stepless speed regulation (5) Nonpolluting (6) Little resistance losing (7) Fire and explosion prevention, high flow rate (8) Working in high temperature.	(1) The gas is easy to be compressed and leak (2) The speed is easy to change under the load (3) It is difficult to precise control, cannot be used under low temperature (4) The gas is difficult to sealed (5) Working pressure is usually smaller than 0.8 Mpa, which only applies to small power driving. Unsuitable for high-power system.	Ankle-foot orthosis of Michigan University, USA

SEA (series elastic actuator) drive		(1) High control precision (2) High security (3) Weaken inertia impaction (4) Reducing the friction losses (5) Storing energy.	(1) Rigidity is restricted by elastic components (2) Large volume (3) Heavy (4) Complicated structure (5) High power.	The Exoskeleton of the Delaware State University [72]

**Table 3:** Overview of training modes for rehabilitation robot.

Training modes	Characteristics	Representative works
Passive mode	The robot helps the patient track the predetermined trajectory through repeated tracking control for passive training.	Ankle robot and gait orthosis [90–92] Gait Trainer (GTI) [30] LOPES [93]

Active mode	When the patient has a certain initiative, the rehabilitation robot will change its trajectory or assistance force.	AAFO [20] LOPES [93] ALEX [17]

Active assist mode	A kind of “active” mode. The patient does not need any help to move the limb. When the threshold value reaches a certain standard, it will trigger the robot.	HAL [22] KAFO [21] G-EO Systems [32]

Active resist mode	A kind of “active” mode. When the patient moves the limb, the robot provides resistance to make the exercise more challenging.	ARBOT [94, 95] Rutgers ankle [25]

**Table 4:** Detection method of human robot interaction information.

HRI	Detection signal	Detection method
pHRI	Kinematics information Force/torque information	Angle sensor, acceleration sensor Pressure sensor, torque sensor
cHRI	Muscle motility information Brain motility information	EMG, sEMG [98] EEG [99]
